# Plasma cell-free DNA methylation analysis for ovarian cancer detection: Analysis of samples from a case-control study and an ovarian cancer screening trial

**DOI:** 10.1002/ijc.34757

**Published:** 2023-10-20

**Authors:** Chiara Herzog, Allison Jones, Iona Evans, Daniel Reisel, Adeola Olaitan, Konstantinos Doufekas, Nicola MacDonald, Angelique Flöter Rådestad, Kristina Gemzell-Danielsson, Michal Zikan, David Cibula, Lukáš Dostálek, Tobias Paprotka, Andreas Leimbach, Markus Schmitt, Andy Ryan, Aleksandra Gentry-Maharaj, Sophia Apostolidou, Adam N Rosenthal, Usha Menon, Martin Widschwendter

**Affiliations:** 1 European Translational Oncology Prevention and Screening (EUTOPS) Institute, Hall in Tirol, Austria; 2 Research Institute for Biomedical Aging Research, Universität Innsbruck, Innsbruck, Austria; 3 Department of Women's Cancer, UCL EGA Institute for Women's Health, University College London, London, UK; 4 Department of Women's and Children's Health, Division of Obstetrics and Gynecology, Karolinska Institutet and Karolinska University Hospital, Stockholm, Sweden; 5 Department of Gynecology and Obstetrics, Charles University in Prague, First Faculty of Medicine and Hospital, Na Bulovce, Czech Republic; 6 Department of Gynaecology, Obstetrics and Neonatology, First Faculty of Medicine, Charles University, Prague and, General University Hospital, Prague, Czech Republic; 7 GENEWIZ Germany GmbH, Bahnhofstraße, Germany; 8 Eurofins Genomics Europe Sequencing GmbH, Konstanz, Germany; 9 MRC Clinical Trials Unit at UCL, Institute of Clinical Trials and Methodology, University College London, London, UK

**Keywords:** cell-free DNA, diagnosis, methylation, ovarian cancer

## Abstract

Analysis of cell-free DNA methylation (cfDNAme), alone or combined with CA125, could help to detect ovarian cancers earlier and may reduce mortality. We assessed cfDNAme in regions of *ZNF154, C2CD4D* and *WNT6* via targeted bisulfite sequencing in diagnostic and early detection (preceding diagnosis) settings. Diagnostic samples were obtained via prospective blood collection in cell-free DNA tubes in a convenience series of patients with a pelvic mass. Early detection samples were matched case-control samples derived from the UK Familial Ovarian Cancer Screening Study (UKFOCSS). In the diagnostic set (n_cases_ = 27, n_controls_ = 41), the specificity of cfDNAme was 97.6% (95% CI: 87.1%-99.9%). High-risk cancers were detected with a sensitivity of 80% (56.3%-94.3%). Combination of cfDNAme and CA125 resulted in a sensitivity of 94.4% (72.7%-99.9%) for high-risk cancers. Despite technical issues in the early detection set (n_cases_ = 29, n_controls_ = 29), the specificity of cfDNAme was 100% (88.1%-100.0%). We detected 27.3% (6.0%-61.0%) of high-risk cases with relatively lower genomic DNA (gDNA) contamination. The sensitivity rose to 33.3% (7.5%-70.1%) in samples taken <1 year before diagnosis. We detected ovarian cancer in several patients up to 1 year before diagnosis despite technical limitations associated with archival samples (UKFOCSS). Combined cfDNAme and CA125 assessment may improve ovarian cancer screening in high-risk populations, but future large-scale prospective studies will be required to validate current findings.

## 1 Introduction

Despite considerable efforts in screening and treatment over the last decades, ovarian cancer (OC) remains the deadliest gynaecological cancer. Among the heterogeneous OC subtypes, high-grade serous ovarian carcinoma (HGSOC) accounts for three quarters of OCs; HGSOC is diagnosed at advanced stages in ~75% of cases^1^ and has <50% 5-year survival rates.^2^ The UK Collaborative Trial of Ovarian Cancer Screening (UKCTOCS) investigated a multimodal screening approach including the risk of ovarian cancer algorithm (ROCA; longitudinal CA125 level assessment) followed by transvaginal ultra-sound in ROCA-positive cases. UKCTOCS reported a significant stage shift, that is, a 10% reduction in advanced stage diagnoses.^3,4^ Unfortunately, this approach did not translate into a survival benefit.^5^ Screening using these modalities can therefore not be recommended in the general population. ROCA-based screening every 4 months has also been shown to result in a larger stage shift in a high-risk population (≥10% lifetime risk for ovarian cancer) in the UK Familial Ovarian Cancer Screening Study (UKFOCSS)^6^ and a recent follow up project—Avoiding Late Diagnosis Ovarian Cancer (ALDO),^7^ but it remains unclear whether this stage shift results in a survival benefit.

While CA125 is the most widely used biomarker for ovarian cancer screening, it can also be detected in the serum of patients with cancers other than ovarian cancer^8^ or in those with benign pelvic conditions.^9^ To address this, in UKCTOCS, a multimodal ovarian cancer screening approach had to incorporate repeat testing (CA125 and transvaginal ultrasound scans) in those who were found to be ROCA positive, to attain the required minimum specificity for general population screening. This resulted in a 30-week (interquartile range, 18-43 weeks) interval between initial ROCA-positivity and surgery in patients for whom the relevant annual CA125 was <35 U/mL.^3^ In addition to protocol-driven repeat testing, a reluctance among clinicians to operate in the absence of a visible tumour on transvaginal ultrasound contributed to this long interval. Currently, transvaginal ultrasound does not have the sensitivity to detect very early lesions as a second line testing. In addition, in the ultrasound arm of the UKCTOCS—where it was used as a primary screening test—ultrasound exhibited low specificity, low positive predictive value and did not result in a stage shift.^10^

The results of 700 000 women years of annual multimodal screening in UKCTOCS provide clear evidence that while CA125 dynamics can attain high sensitivity in ovarian cancer screening (86%), there is an urgent need for it to be combined with an independent, highly specific test. A non-invasive blood marker would be ideal. New technologies assessing material shed by tumours, including cell-free DNA (cfDNA) mutations harbouring cancer-derived structural or single nucleotide variants, could improve sensitivity and specificity of testing. Nonetheless, approaches investigating mutations in cfDNA are complicated by contamination or confounding signals from white blood cells (such as clonal haematopoiesis)^11–13^ and small relative differences between cases and controls requiring deep sequencing to improve low signal-to-noise ratios.^14–16^

Cell-free DNA methylation (cfDNAme) analysis is emerging as a promising biomarker for cancer screening.^17,18^ DNA methylation changes occur early during cancer development,^19–21^ are more frequently observed than somatic mutations, and may offer an improved signal-to-noise ratio. Via epigenome-wide approaches we have previously identified regions that may be suitable for detection of ovarian cancers and their discrimination from benign conditions.^18^ Here, we report the sensitivity and specificity of a multiplexed targeted bisulfite sequencing assay in two scenarios: diagnosis of ovarian cancers in patients with clinically identified ovarian masses and earlier detection in high-risk patients prior to the detection of a pelvic mass in the UKFOCSS trial. Moreover, we evaluate the analytical stability of the assay in a set of samples collected during the day or during the night, given recent reports of increased release of tumour material, including both cells^22^ and cell-free DNA,^23,24^ during the resting phase.^22^

## 2 Materials and Methods

### 2.1 Study design

#### 2.1.1 Diagnostic set

Samples in the diagnostic set were collected across three European recruitment sites (University College London Hospital [UCLH] [UK], Charles University Hospital [Czech Republic], and the Karolinska Institutet [Sweden]). Samples were collected between September 2019 and December 2020. Potential participants presenting to relevant clinics were approached by participating doctors and study nurses. Prior to taking part, each potential study volunteer was given a Participant Information Sheet and a Consent Form and the rationale for the study was explained. Women over the age of 18 years who consented to participate in the study and agreed to donate a blood sample were eligible to participate. Participants with a history of prior cancers (excluding melanoma) and those who had started treatment (surgery, radio- or chemotherapy or other local or systemic treatments) were excluded. For samples collected at UCLH, exact timing from diagnosis to sample collection was not recorded, but blood sample collection generally occurred on the morning of surgery which typically happened within 2 to 4 weeks after initial diagnosis. At Charles University Hospital and the Karolinska Institutet, samples were collected at initial clinical diagnosis which happened generally 1 to 4 weeks before pathological confirmation and surgery. Biological samples were pseudoanonymised using a participant study number. Each recruitment site maintained a securely stored file linking personal identifiers to the study number.

**Figure 1:**
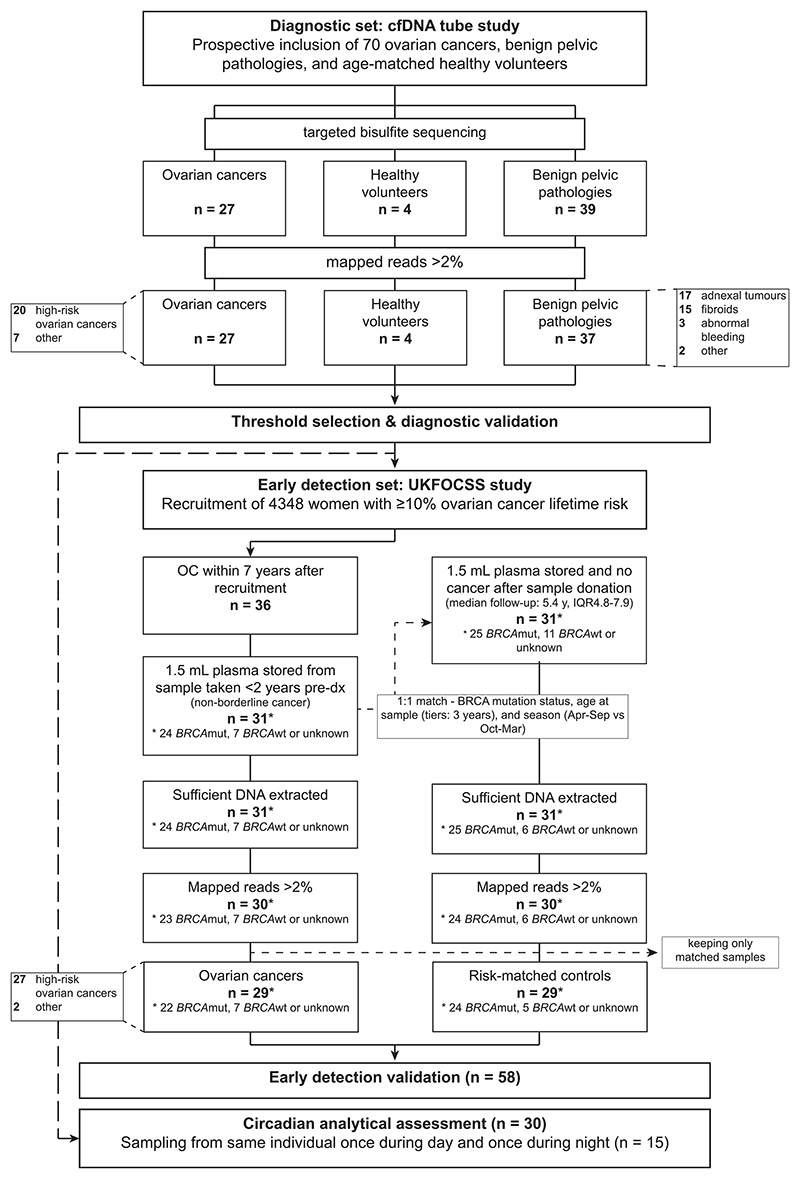
Overview of the study. High-risk cancers included all grade 2 and 3 cancers.

Peripheral blood (4 mL) was collected in Roche CE-IVD cell-free DNA collection tubes (#07785666001). After blood collection, samples were processed within 24 h locally to plasma according to the manufacturer's instructions. Plasma was aliquoted in cryovials and stored at −80°C and shipped to Eurofins Genomics Europe Sequencing GmbH, Konstanz, Germany, on dry ice for further processing. An overview of participant and sample flow is shown in Figure 1 and the STARD (Standards for Reporting Diagnostic accuracy,^25^ Figure S1). Detailed participant characteristics are reported in Table 1.

#### 2.1.2 Early detection set

Samples in the early detection set were collected as part of the UK Familial Ovarian Cancer Screening Study (UKFOCSS). UKFOCSS was an intervention trial which aimed to establish the performance of ovarian cancer screening with longitudinal serum CA125 measurements, interpreted using the ROCA and transvaginal sonography for women at high risk of ovarian cancer or fallopian tube cancer. Entry criteria and recruitment are detailed in previous publications.^6,26^ Blood samples were collected in 9 mL Greiner Bio One KE3DTA (cat #455036) between June 14, 2007 and May 15, 2012. Samples were stored in liquid nitrogen between June 2007 and November 2021, when straw retrieval was actioned from an offsite repository and samples were shipped to Eurofins Genomics Europe Sequencing GmbH, Konstanz, Germany. Among the UKFOCSS study population, consisting of high-risk women with an estimated minimum 10% lifetime risk for ovarian cancer, 36 non-borderline ovarian cancers were identified after 7 years of follow-up. Cases with approximately 1.5 mL plasma samples were matched 1:1 with controls who had remained cancer-free until the end of follow-up (median follow-up 5.4 years [4.8-7.9]) and included in this study (Figure 1, STARD Figure S2). When a sample did not pass sequencing quality control (QC), its matched sample was also excluded from the analysis. Detailed participant characteristics are reported in Table 1.

#### 2.1.3 Circadian analytical assessment set (‘Precision set’)

Samples to evaluate analytical stability of the assay in relation to circadian rhythms were collected at Charles University Hospital (Czech Republic). Participants included 15 ovarian cancer cases. Peripheral blood (4 mL) was collected in Roche CE-IVD cell-free DNA collection tubes (#07785666001). Diurnal samples were collected between 8:00 and 18:00, while nocturnal samples were collected between the hours of 02:00 and 04:00 prior to surgery. The minimum time between sampling of the diurnal and nocturnal sample was 8 h. After blood collection, samples were processed within 24 h locally to plasma according to the manufacturer's instructions. Plasma was aliquoted in cryovials and stored at −80°C and shipped to Eurofins Genomics Europe Sequencing GmbH, Konstanz, Germany, on dry ice for further processing. Detailed participant characteristics are reported in Table S1.

### 2.2 DNA extraction, bisulfite conversion and targeted sequencing

**Figure 2:**
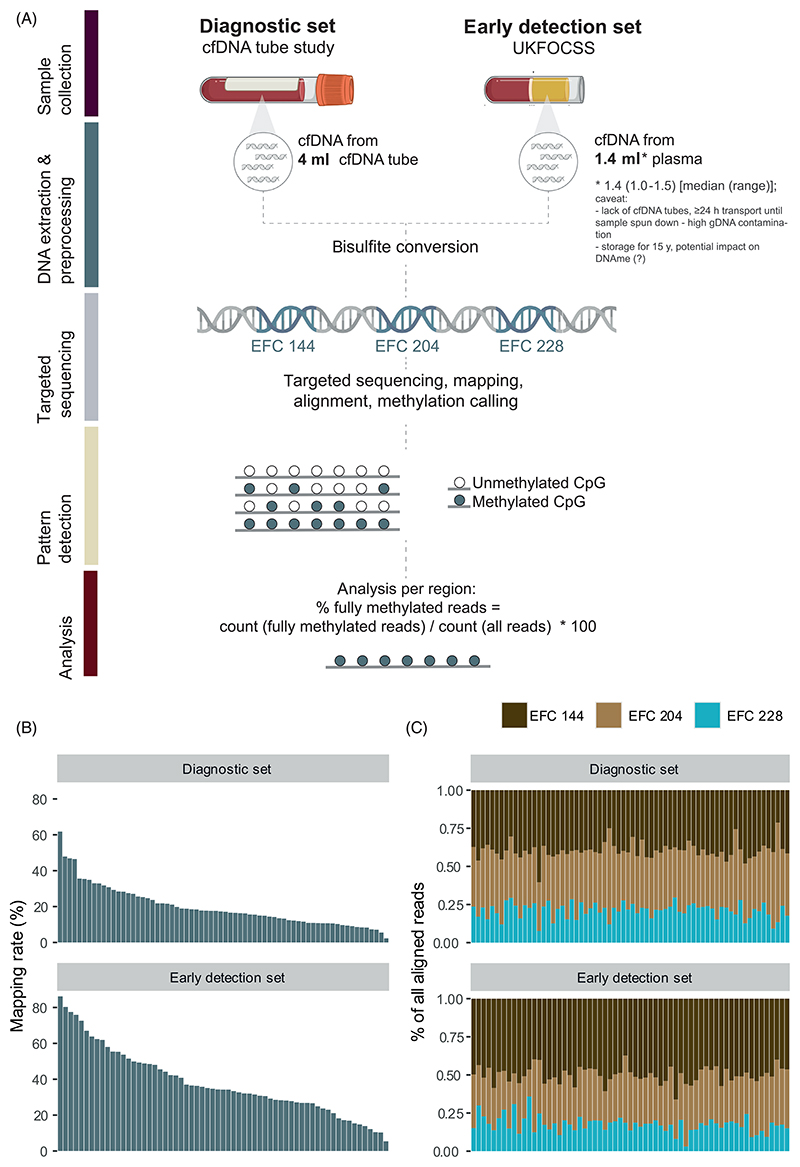
Cell-free DNA methylation pipeline and quality control metrics. (A) Overview of sample processing and analysis. (B) Visualisation of mapping rates for samples in the Diagnostic and Early detection sets. (C) Proportion of aligned reads in each of the three targeted regions (EFC 144, EFC 204, EFC 228).

An overview of the sample workflow for cfDNAme analysis is shown in Figure 2. DNA was processed and sequenced at Eurofins Genomics Europe Sequencing GmbH (Konstanz, Germany). Briefly, DNA was isolated from the plasma samples using the QIAamp Circulating Nucleic Acid Kit (Qiagen, Cat. #55114) and quantified with the Agilent Fragment Analyser and the High Sensitivity Large Fragment Analysis Kit (Agilent, USA). DNA was bisulfite converted with the EZ DNA Methylation-Lightning Kit (Zymo Research, USA). Targeted bisulfite sequencing libraries were prepared at Eurofins Genomics Europe Sequencing GmbH. Bisulfite modification was performed with 1 to 4 mL plasma equivalent. A multiplex PCR approach was used to amplify the corresponding targets, which were then converted to an Illumina-compatible library (Eurofins Genomics Europe Sequencing GmbH). Ultra-high coverage sequencing was performed on Illumina's NovaSeq6000 with 150 bp paired-end mode, respectively.

### 2.3 Methylation calling and cell-free DNA methylation score

Methylation was called using bismark v0.22.3,^27^ aligning to target regions EFC 144, EFC 204 and EFC 228 (Table S2). For each region and read, the methylation status of each CpG was analysed. Any samples with a mapping rate below 2% were excluded (Figure 1). No other filtering steps were applied. The percentage of fully methylated reads (ie, reads in which all CpGs were methylated) out of all reads in the region was then computed. A summary of read statistics is shown in Table S3, and sample-level read statistics are provided in Table S4.

The WID-cfOC (Women's cancer risk IDentification—cell-free DNA methylation for Ovarian Cancer) was derived as follows: for each region, a % fully methylated reads threshold value at 97% specificity was defined in the Diagnostic set. If values in one or more regions were above their respective thresholds, the sample counted as WID-cfOC positive. The WID-cfOC was then applied in both the Diagnostic and Early detection set.

### 2.4 Genomic DNA (gDNA) contamination analysis

Genomic DNA (gDNA) contamination in UKFOCSS plasma samples was quantified as the ratio of the calculated amount of cfDNA relative to the amount of gDNA (Figure S3). The median cfDNA/gDNA ratio was computed in all samples and samples were stratified into a higher (cfDNA/gDNA ratio ≤ median) and lower gDNA contamination group (cfDNA/gDNA ratio > median), respectively (Figure S3C,D).

### 2.5 CA125 analysis and threshold

CA125 analysis was conducted by The Doctor's Laboratory, London (cfDNA tube study) using the cobas Elecsys CA 125 II kit, as part of the UKFOCSS study (centrally or locally), as previously described.^6,26^ CA125 results ≥35 units/mL were classified as abnormal.^28^ CA125 data in the UKFOCSS study were not always available from the same time as the sample on which cfDNA was analysed (available for n = 46, including n = 22 controls, n = 24 ovarian cancer cases). For the remaining 12 samples, CA125 measurements were obtained from health records before or after the index sample was taken where possible, with the following assumptions being made based on the closest CA125 measurement to index sample collection: if the CA125 score was positive (≥35 units/mL) before index sample collection, CA125 was assumed positive; if CA125 results after index sample collection were negative (<35 units/mL), CA125 was assumed to be negative in the index sample. Any samples without CA125 information (n = 1), with negative scores in the time before index sample collection (n = 2), or with positive scores in the time after index sample collection (n = 2) were excluded from the CA125 analysis as the CA125 score at index sampling could not be accurately inferred. Seven samples met the additional time-difference criteria for CA125.

### 2.6 Statistical analysis

All analyses were conducted in R 4.3.1 (2023-06-16). Plots were generated using ggplot2 (3.4.3). Area under the receiver operating characteristic curves were created using pROC (1.18.4). Confidence intervals for sensitivity and specificity were calculated using the Clopper-Pearson method, implemented in the epiR package (2.0.63). Confidence intervals for difference in sensitivity and specificity were calculated as follows:

$$IC_{0,95}=\text{Δ}\pm t_{\alpha /2}\frac{S_{D}}{\sqrt{N}},$$

where Δ is the mean sample difference, S_D_ is the SE for the difference scores, N is the number of difference scores, and t_α/2_ is the critical value of the *t* distribution for a two-tailed test with *p* < α and N – 1 degrees of freedom. Analyses were stratified by distinction between all ovarian cancers and high-risk ovarian cancers, which were defined as all grade 2 and 3 cancers.

## 3 Results

### 3.1 A cfDNAme score consisting of three regions, the WID-cfOC, identifies ovarian cancers

**Figure 3:**
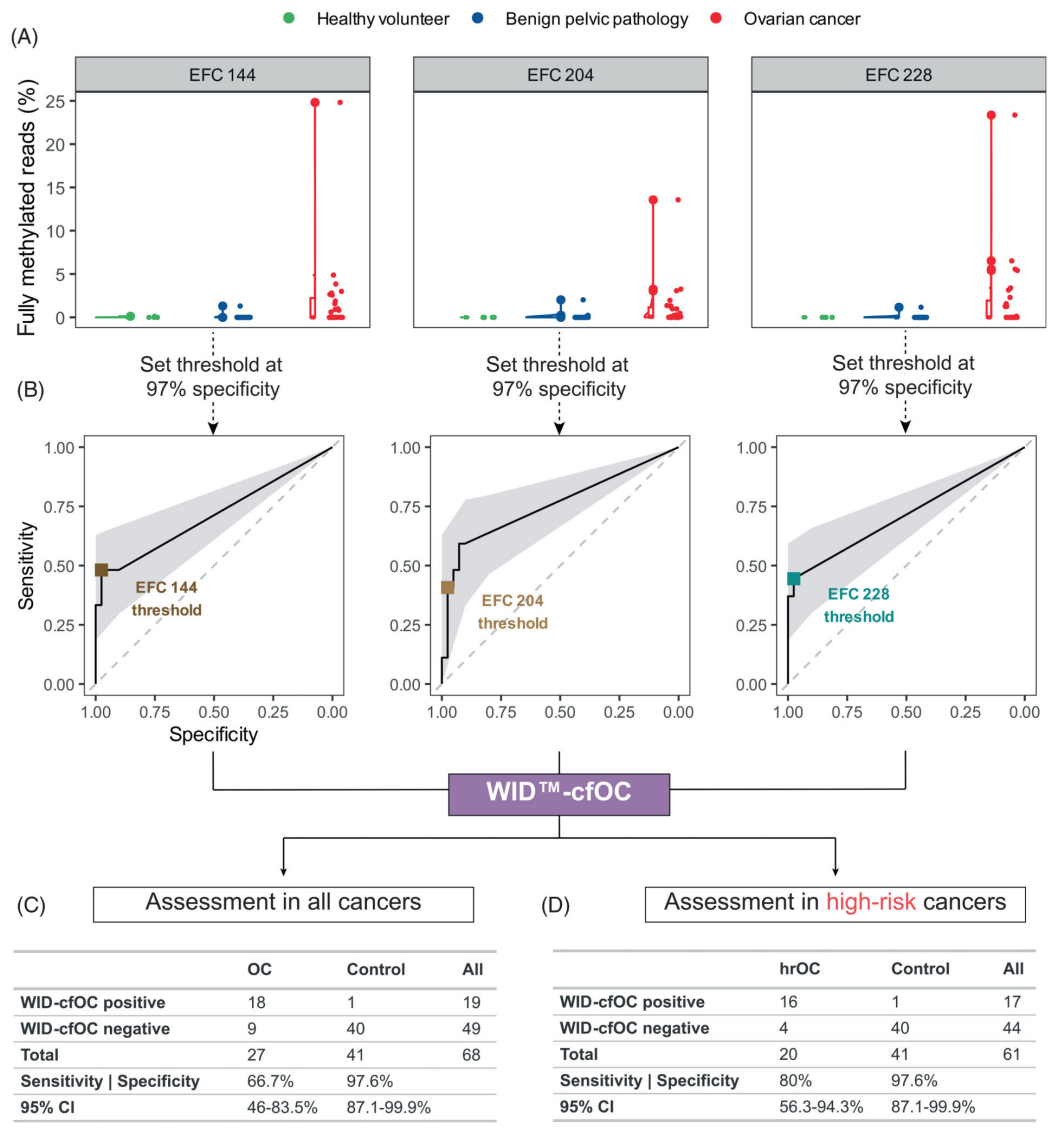
Threshold selection and diagnostic validation. (A) Values of fully methylated reads (% of all reads in each respective region) for the three targeted regions EFC 144, EFC 204 and EFC 228 in healthy volunteers and women with benign pelvic pathologies or ovarian cancer. (B) Receiver operating characteristic curves for each of the four regions. A specificity cutoff of 97% and the corresponding value of fully methylated reads was set as the threshold. A cfDNAme score (WID-cfOC) was derived from regions EFC 144, EFC 204 and EFC 228. If any of the regions was above the respective threshold, the WID-cfOC was regarded positive. The WID-cfOC was evaluated in (C) all ovarian cancers and (D) high-risk ovarian cancers (see Table 1).

A summary of study participants is shown in Table 1. In the diagnostic set, we analysed cfDNAme patterns in 27 ovarian cancer cases, four healthy volunteers, and 37 women with benign pelvic pathologies via targeted bisulfite sequencing of the three regions EpiFem-Care (EFC) 144, EFC 204 and EFC 228 located in the genes *ZNF154, C2CD4D* and *WNT6*, respectively (Figure 2 and Table S2). The percentage of fully methylated reads is shown in Figure 3A. We selected cutoffs for the WID-cfOC score by identifying the value (% of fully methylated reads) closest to 97% specificity on the area under the receiver operating characteristic curve for each region (Figure 3B). If the value for any of the three regions was above the pre-specified respective threshold, the score was positive. Only if all values in all three regions were negative, the score was negative. The specificity of the WID-cfOC score was 97.6% (95% CI: 87.1%-99.9%) with a sensitivity of 66.7% (46%-83.5%) and 80% (56.3%-94.3%) for detection of all and high-risk cancers (grade 2/3 cancers), respectively (Figure 3C, D).

For 63 individuals, both WID-cfOC and CA125 data were available (Figure S4A; n = 24 OCs, n = 30 controls). In high-risk cancers in this group (n = 18 cases), cfDNAme WID-cfOC alone exhibited a slightly lower sensitivity (77.8% [95% CI: 52.4-93.6%] vs 83.3% [95% CI: 58.6-96.4%]), but a higher specificity than CA125 (97.4% [95% CI: 86.5-99.9%] vs 87.2% [95% CI: 72.6-95.7%]). Combining cfDNAme and CA125, that is, counting women positive that were either WID-cfOC or CA125-positive, increased the sensitivity for high-risk cancers compared to either marker alone to 94.4% (95% CI: 72.7%-99.9%), albeit at a loss of specificity compared to the WID-cfOC score alone (87.2%, 95% CI: 72.6%-95.7%) (Figure S4A,B and Table S5). The differences were not significant, as shown by the confidence interval of difference in sensitivity and/or specificity, which is likely attributed to the relatively low number of samples. The WID-cfOC score detected two out of three of CA125-negative high-risk cancers (Figure S4D). Only one high-risk cancer (HGSOC) remained undetected. The remaining double negative cancers were all low-grade cancers (Figure S4C). In CA125-positive samples, the WID-cfOC score maintained high specificity and sensitivity (Figure S4E, F).

### 3.2 cfDNAme-based identification of future ovarian cancer cases

We assessed the suitability of the WID-cfOC score to identify cancers in samples predating diagnosis. Plasma samples (median volume: 1.4 mL, stored for up to 15 years) from women with a ≥10% lifetime risk of developing ovarian cancer, including 29 OC cases and 29 cancer-free matched controls, were subjected to the targeted bisulfite sequencing assay. Mapping rates and the distribution of reads per region are shown in Figure 2B, C. As expected, because samples were not collected with tubes specific for cfDNA extraction and underwent long-term storage, some samples showed considerable contamination with genomic DNA (gDNA) that was likely caused by lysis of white blood cells (Figure S3). Analysis was therefore stratified by gDNA contamination (see Section 2).

**Figure 4:**
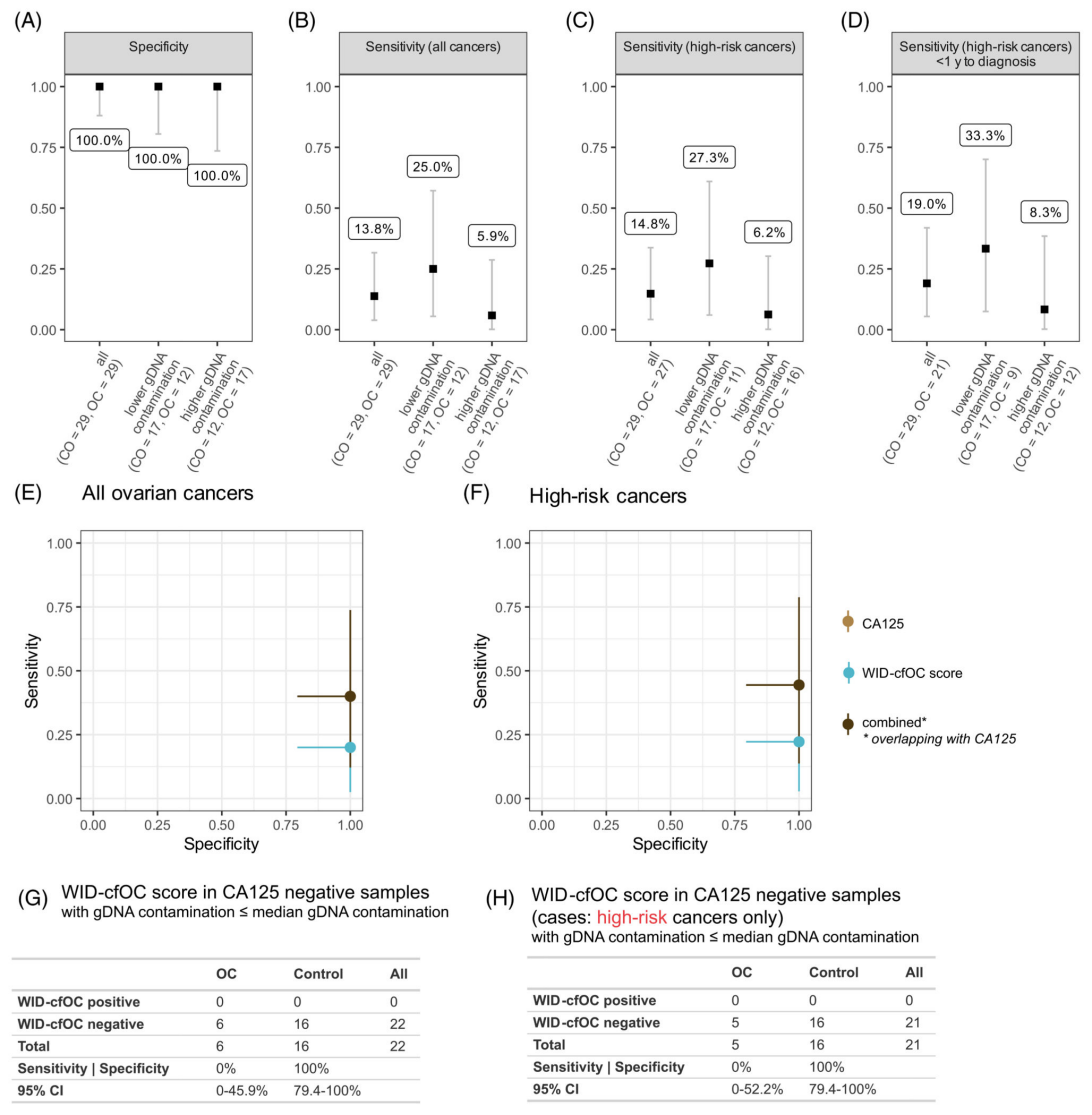
Sensitivity and specificity of the cfDNAme score in an early detection setting. All measures are demonstrated in all samples, or those with lower or higher gDNA contamination, respectively. gDNA contamination was quantified via cfDNA/gDNA peaks and a ratio was calculated (see Figure S3). Samples with a higher than median cfDNA/gDNA ratio had lower gDNA contamination and vice versa. (A) Specificity of the cfDNAme score in UKFOCSS samples. (B) Sensitivity for the cfDNAme score in all ovarian cancer patients, (C) samples from high-risk ovarian cancer patients, or (D) samples from high-risk ovarian cancer patients collected <1 year from diagnosis. For samples with matched CA125 data, cfDNAme, CA125 and a combined score (positive when either cfDNAme or CA125 were positive) were evaluated in (E) all cancers or (F) high-risk cancers regardless of gDNA contamination. CA125 and combined score overlap, likely due to a limited amount of data based on a small sample size. (G, H) Evaluation of sensitivity and specificity in CA125 negative samples including all or only high-risk cancers.

The WID-cfOC score exhibited 100.0% specificity regardless of gDNA contamination (Figure 4A). Sensitivity was dependent on gDNA contamination: 13.8% of all cancers were identified (Figure 4B); 25.0% of cancer samples with a lower than median gDNA contamination were identified, and 5.9% of samples with higher gDNA contamination. Sensitivity of the WID-cfOC score was improved for high-risk cancers with lower gDNA contamination (27.3%, Figure 4C), and further improved to 33.3% for high-risk cancers <1 year to diagnosis (Figure 4D). When restricting the analysis to *BRCA1/2* mutation carriers, sensitivity was further improved, but sample numbers for non-*BRCA1/2* mutation carriers were limited (Figure S5).

For n = 46 samples (22 controls, 24 cancers), CA125 data was available at the time of sampling. We evaluated cfDNAme alone, CA125 alone, or a combined score in 26 of these samples that exhibited a lower than median gDNA contamination (Figure 4 and Table S6). In high-risk cancers, cfDNAme exhibited a sensitivity of 22.2% (95% CI: 2.8%-60.0%) and a specificity of 100.0% (95% CI: 79.4%-100.0%). CA125 testing exhibited a sensitivity of 44.4% (95% CI: 13.7%-78.8%) and specificity of 100.0% (95% CI: 79.4%-100.0%). For these samples, the WID-cfOC score did not improve sensitivity over CA125 alone and the combined score resulted in a sensitivity of 44.4% (95% CI: 13.7%-78.8%) and specificity of 100.0% (95% CI: 79.4%-100.0%) (Figure 4E-H and Table S6).

### 3.3 WID-cfOC score and cancer stage

We evaluated the detection of cancers using cfDNAme and CA125 based on cancer stage, albeit limited numbers of early-stage cancer samples were available in our datasets. Within the diagnostic set, neither test identified any stage I cancers, but a stage II cancer was correctly identified (Table S7). One out of two stage II cancers was correctly identified in the early detection set (samples with lower than median gDNA contamination). Most cancer cases in higher stages (III and IV) were detected in the diagnostic set (8/9 stage III cancers, 6/6 stage IV cancers), and 3/5 stage III cancers were detected in the early detection set.

### 3.4 Analytical validation of circadian stability of the assay

To evaluate the analytical stability of our assay across different circadian stages, we assessed cfDNAme levels in samples collected from the same individuals with ovarian cancer (n = 15) during the day (diurnal sample, between 08:00 and 18:00) and during the consecutive night (between 02:00 and 04:00), prior to surgery (Figure S6). Interestingly, the cfDNA/gDNA ratio was higher at night, indicating a higher cfDNA yield (Figure S6A). The agreement between values was generally high (Figure S6C, >0.77) but tended to be higher for values above 1% fully methylated reads (Pearson's correlation coefficient for all three regions = 0.99, Figure S6D) than below or equal 1% (Pearson's correlation coefficient: EFC 144 = –0.31, EFC 204 = –0.25, EFC 228 = 0.16, Figure S6E). EFC 228 was involved in all 5/15 discrepant calls (no amplification was found in ‘negative’ samples).

## 4 Discussion

Here we describe the sensitivity and specificity of targeted cfDNAme analysis for detection of ovarian cancer. cfDNAme offers a high signal-to-noise ratio and our analysis in a case-control setting indicates it may enable improved specificity with similar sensitivity to CA125 for detection of high-risk ovarian cancers in women with a pelvic mass. The combination of CA125 and cfDNAme in the diagnostic setting improved (albeit not significantly, likely due to low numbers) the sensitivity compared to either test alone, detecting 94.4% of high-risk ovarian cancers at a specificity of 87.2%.

In the early detection UKFOCSS setting, cfDNAme exhibited a specificity of 100% but sensitivity was low. cfDNAme sensitivity likely suffered as the samples were not collected with the intent of cfDNAme analysis and stored for up to 15 years prior to analysis, resulting in variable levels of genomic DNA contamination. Moreover, cfDNAme analysis typically requires large amounts of blood (up to 12 mL),^29^ but only limited material was available for our analysis (median 1.4 mL). Despite these challenges, the WID-cfOC score detected 33.3% of high-risk cancers in samples with lower gDNA contamination predating diagnosis by up to 1 year. One may speculate that the signal could have been improved with more suitable sample collection and storage conditions for the purpose of cfDNAme analysis, although this can only be conclusively demonstrated in prospective studies. In addition, in 20% (12/58) of women the CA125 levels were not measured at the same time as the plasma samples that were assayed in this study. For the comparison of the performance of CA125 or cfDNAme alone, we therefore included CA125 results that were measured in the previous or subsequent sample (see Section 2). Overall, our data provide an initial insight into the potential of combining CA125 and cfDNAme, although our conclusions are limited by the small sample size (Figure 4).

**Figure 5:**
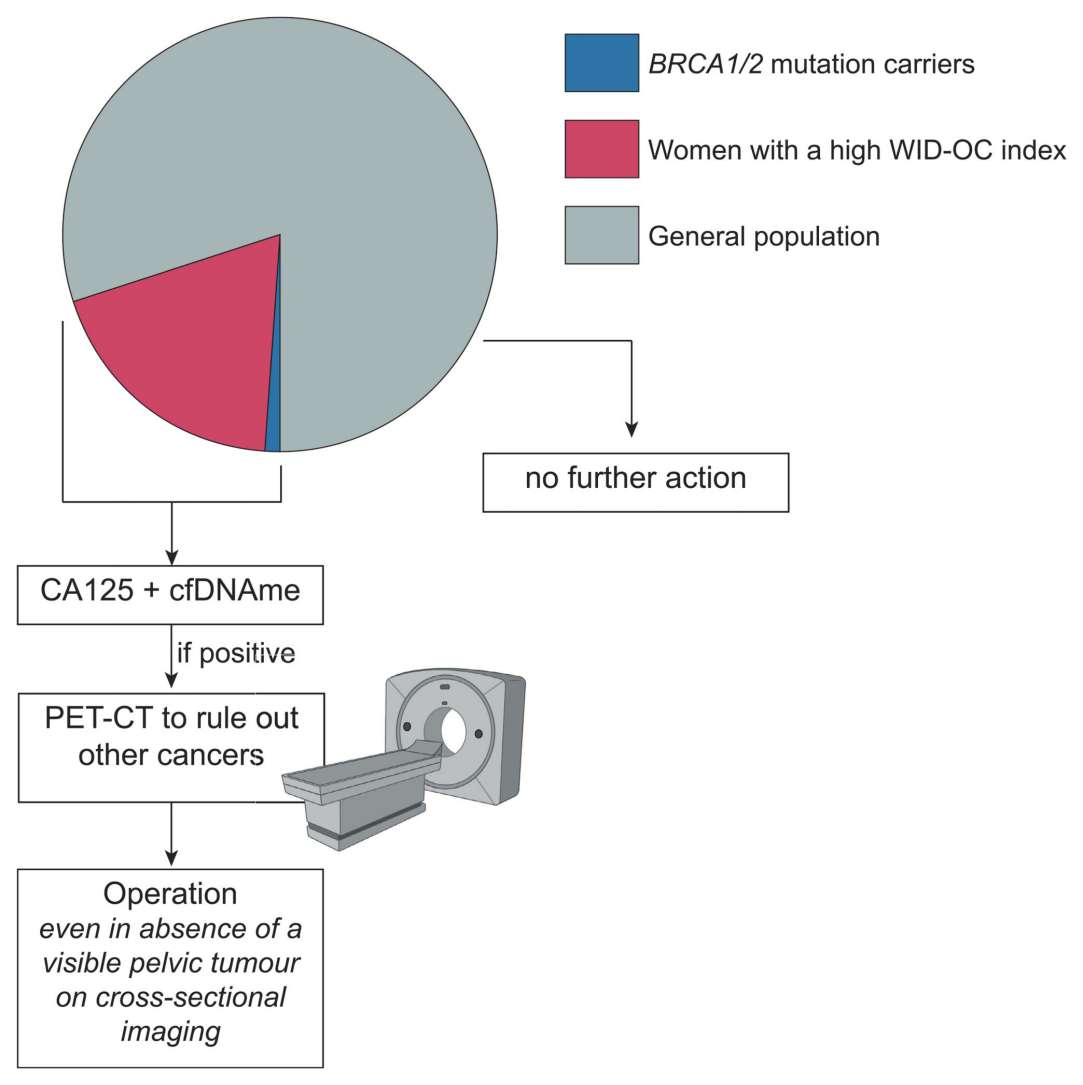
Outlook on ovarian cancer screening using combined molecular tests.

To advance the field of early detection, tumours should arguably be detected before they reach a size that is visible on cross-sectional imaging. Application of combined cfDNAme and CA125 testing in individuals at increased risk may help to enable more sensitive, earlier detection of ovarian cancers. Women at high risk could include those with germline *BRCA1/2* mutations or individuals identified by novel tests, such as the Women's Cancer Risk Identification index for ovarian cancer (WID-OC index), which is a measure recently shown to predict the presence of ovarian cancer.^30^ PET-CT imaging could be conducted in these high-risk women to rule out presence of other cancers. Due to the relatively high specificity, particularly for double CA125/cfDNAme positive women, an operation could be scheduled even in the absence of a visible mass on imaging (Figure 5) following further validation of the current test or other promising candidates.^31–33^ Of note, the current study is one of the few that describe cfDNA methylation prior to current diagnosis.^18^ Lu et al recently described the discovery of regions of interest using cell-free methylated DNA immunoprecipitation and high-throughput sequencing (cfMeDIP-seq) and achieved high accuracy of their regions in a hold-out 20% test set but did not perform cross-population validation in an independent dataset. Marinelli et al and Liang et al did perform independent validation, but not validate their method in samples predating diagnosis. Future systematic reviews (such as^34^), meta-analyses and benchmarking studies validating several promising cfDNA markers across the same patient population should be encouraged where technically feasible to identify the most promising candidates for early detection for urgent clinical prioritisation.

A recent study described circadian dependence in tumour cell intra-vasation, with enhanced release of tumour cells during the resting phase,^22^ but the literature on diurnal variation in cfDNA levels is conflicting.^23,24^ We observed significantly higher ratio of cfDNA/gDNA in samples collected during the night (*P* = .0084 in paired Wilcoxon test), indicating potential higher levels of cfDNA during the night. Nonetheless, our results indicated high stability of values, although agreement was higher in values above 1% fully methylated reads, consistent with the notion that the assay may be more robust with higher values of fully methylated reads. Such observations must be considered for further analytical validation of the assay, as they can ultimately influence WID-cfOC positive/negative calls (Figure S6D). Future studies will be required to optimise analytical precision while maintaining high sensitivity in the context of low abundance cfDNA.

Our study suffers from several limitations, including those inherent to a case-control design. To improve generalizability, we have included age- and risk-matched controls and selected samples from a large prospective screening study (UKFOCSS). Our conclusions are further limited by a small sample size, resulting in a limited representation of various histological subtypes and low stages, and, in the case of the early detection UKFOCSS setting, suboptimal sample processing for cfDNAme analysis. A strength of the current study is the inclusion of control women with pelvic mass in the diagnostic set. CA125 is known to be elevated in certain benign conditions,^9^ and thus displayed, as expected, a lower specificity compared with the WID-cfOC. Future large-scale studies will need to investigate whether in women at high risk for ovarian cancer, the combined analysis of cfDNAme and CA125 leads to a stage shift at an acceptable high positive predictive value.

In summary, our results show the test effectively distinguished benign pelvic conditions and ovarian cancers, and combination of cfDNAme with single CA125 measurement improved sensitivity beyond that of either marker alone. The WID-cfOC score identified 33.3% of high-risk (grade 2 or 3) ovarian cancers up to a year before diagnosis. Lastly, scores of fully methylated cfDNAme values (%) exhibited limited variability between samples taken during the day and during the night, although some differences in scores warrants further investigation of analytical precision and its dependence on circadian rhythms. The combination of the WID-cfOC with CA125 may be suitable to improve cancer detection in high-risk populations.

## Supplementary Material

Supp 1

Table 1

Table 2

## Figures and Tables

**Table 1:** Participant characteristics in the diagnostic and early detection sets.

	Diagnostic set (cfDNA tube study)			Early detection set (UKFOCSS)	
Characteristic	Control n = 41	Ovarian cancer n = 27		Control n = 29	Ovarian cancer n = 29
Detailed pathology, n (%)					
Healthy volunteer (none)	4 (9·8)			29 (100)	
Abnormal bleeding	3 (7·3)				
Adnexal tumour	17 (41)				
Fibroids	15 (37)				
High-grade serous ovarian cancer		19 (70)			22 (76)
Low-grade serous ovarian cancer		3 (11)			
Endometrioid ovarian cancer		1 (3·7)			4 (14)
Mucinous ovarian cancer		1 (3·7)			
Other^a^		3 (11)			1 (3·4)
Unknown	2 (4·9)				2 (6·9)
Age at sample taken, Median (IQR)	57 (48-66)	58 (52-64)		49 (43-60)	49 (46-57)
Age at diagnosis, Median (IQR)					51 (47-57)
Time to event (days), Median (IQR)^b^				1994 (1764-2885)	1793 (1673-2177)
Grade (cases), n (%)					
1		5 (20)			1 (3·6)
2		1 (4·0)			
3		19 (76)			27 (96)
Risk classification (cases), n (%)^c^					
Low/unknown		7 (26)			2 (6·9)
High		20 (74)			27 (93)
Stage (cases), n (%)					
I		3 (14)			7 (27)
II		1 (4·8)			2 (7·7)
III		10 (48)			15 (58)
IV		7 (33)			2 (7·7)
CA125 measurement available, n (%)	39 (95)	24 (89)		26 (90)	27 (93)
CA125 measurement time difference (days), Mean (Minimum–Maximum)	0 (0–0)	0 (0–0)		52 (0-1018)	–2 (–118 to 81)
CA125 (U/mL), Median (IQR)^d^	14 (10-22)	152 (42-296)		12 (9-14)	34 (12-69)
*BRCA* mutation status, n (%)					
*BRCA1* mutation carrier				18 (62)	17 (59)
*BRCA2* mutation carrier				6 (21)	5 (17)
Confirmed *BRCA1/2* wild type				4 (14)	6 (21)
Unknown	41 (100)	27 (100)		1 (3·4)	1 (3·4)

a Diagnostic set: one fallopian tube carcinoma, one stage I granulosa tumour and one squamous cell carcinoma; Early detection set: one papillary serous carcinoma of unknown grade.

b Time to event in controls: time to censoring (end of follow-up); in cases: time to diagnosis.

c Grade 2 and 3 cancers were classified as high risk, all others as low risk.

d CA125 information available at the time of blood sampling for 22/29 controls and 24/29 ovarian cancer cases. For the remaining cases, it was available before or after blood sampling (see Section 2).

## Data Availability

Code for the analyses is available at https://github.com/chiaraherzog/WID-cfOC_code. The other data that support the manuscript are available from the corresponding author upon reasonable request.

## What’s new?

Our findings indicate that combining cell-free DNA methylation analysis in three genetic regions with CA125 may improve the sensitivity of ovarian cancer detection in high-risk women. Women with a positive score in either diagnostic modality, particularly double-positive individuals, could be referred for a PET-CT scan to rule out a positive result due to a different cancer, and could undergo surgery even in the absence of a visible ovarian tumour on imaging given the high specificity of cfDNAme.

## Abbreviations

cfDNA: cell-free DNA
DNAme: DNA methylation
EFC: EpiFemCare project
gDNA: genomic DNA
PET-CT: positron emission tomography-computed tomography
ROCA: risk of ovarian cancer algorithm
STARD: Standards for Reporting Diagnostic accuracy
WID-cfOC: Women's cancer risk IDentification-cell-free DNA methylation for Ovarian Cancer.
