# Supplementary material for: Plasma cell-free DNA methylation analysis for ovarian cancer detection: Analysis of samples from a case-control study and an ovarian cancer screening trial
Source: Int J Cancer. Author manuscript; Available in PMC 2025 Jan 27. (PMC7617350; doi:10.1002/ijc.34757)
Supplement: Supp 1 [file EMS202206-supplement-Supp_1.pdf]

Plasma cell-free DNA methylation analysis for ovarian cancer detection – analysis of samples from a case-control study and an ovarian cancer screening trial

Supplementary Material

Chiara Herzog, Allison Jones, Iona Evans, Daniel Reisel, Adeola Olaitan, Konstantinos Doufekas, Nicola MacDonald, Angelique Flöter Rådestad, Kristina Gemzell-Danielsson, Michael Zikan, David Cibula, Lukáš Dostálek, Tobias Paprotka, Andreas Leimbach, Markus Schmitt, Andy Ryan, Aleksandra Gentry-Maharaj, Sophia Apostolidou, Adam Rosenthal, Usha Menon, and Martin Widschwendter

Contents

Supplementary Tables 3

1 Supplementary Table 1. Participant characteristics in the circadian analytical assessment ('precision') set. 3

2 Supplementary Table 2. Details of targeted regions (available as separate file). 4

3 Supplementary Table 3. Summary sequencing statistics for each set. 5

4 Supplementary Table 4. Sample-level sequencing statistics of targeted regions (available as separate file). 6

5 Supplementary Table 5. Sensitivity and specificity of the WID™-cfOC score, CA125, or combination of both in samples in the diagnostic set for which CA125 were available. 7

6 Supplementary Table 6. Sensitivity and specificity of the WID™-cfOC score, CA125, or combination of both in samples in the early detection set samples with lower than median gDNA contamination for which CA125 were available. 8

7 Supplementary Table 7. Evaluation of combined WID™-cfOC and CA125 scoring by stage. 9

Supplementary Figures 10

8 Supplementary Figure 1. STARD Diagram of the diagnostic set (cfDNA tube study). 10

9 Supplementary Figure 2. STARD Diagram of the early detection set (UKFOCSS). 11

10 Supplementary Figure 3. Quantification of genomic DNA (gDNA) contamination. 12

11 Supplementary Figure 4. Comparison of CA125 and cfDNAme score in the Diagnostic set. 13

12 Supplementary Figure 5. Sensitivity and specificity by *BRCA1/2* mutation status in the early detection set. 14

13 Supplementary Figure 6. Precision of the assay as evaluated xin a circadian analytical assessment set. 16

Supplementary Tables

Supplementary Table 1. Participant characteristics in the circadian analytical assessment ('precision') set.

Characteristic	Precision set<br>Ovarian cancer<br>n = 15
**Detailed pathology, n (%)**	
High-grade serous ovarian cancer	13 (87)
Endometrioid ovarian cancer	2 (13)
**Grade (cases), n (%)**	
3	15 (100)
**risk, n (%)**	
**Stage (cases), n (%)**	
**Instance of cancer, n (%)**	
primary	7 (47)
primary (after NACT, prior IDS)	2 (13)
recurrent	6 (40)

Abbreviations: NACT, neo-adjuvant chemotherapy; IDS: interval debulking surgery.

Supplementary Table 2. Details of targeted regions (available as separate file).

(See .xlsx file)

Supplementary Table 3. Summary sequencing statistics for each set.

Characteristic	Diagnostic set<br>n = 68[1]	Early detection set<br>n = 58[1]	Precision set<br>n = 30[1]
**Total reads**	12,705,521 (7,143,124, 16,877,674)	3,676,107 (2,099,901, 6,132,509)	5,098,107 (3,194,765, 6,061,401)
**Reads after filtering**	17 (11, 25)	35 (25, 50)	62 (42, 72)
**Median CpG methylation**	5.5 (4.6, 7.3)	2.0 (1.6, 2.6)	3.2 (2.1, 12.1)
**Bisulfite conversion efficiency**	99.40 (99.30, 99.50)	99.60 (99.50, 99.60)	99.50 (99.50, 99.58)

[1] Median (IQR)

Supplementary Table 4. Sample-level sequencing statistics of targeted regions (available as separate file).

(See .xlsx file)

Supplementary Table 5. Sensitivity and specificity of the WID™-cfOC score, CA125, or combination of both in samples in the diagnostic set for which CA125 were available.

	**All cancers (95% CI)**<br>24 cancers<br>39 controls	**High-risk cancers (95% CI)**<br>18 cancers<br>39 controls
**WID™-cfOC**		
Sensitivity	66.7% (44.7-84.4%)	77.8% (52.4-93.6%)
Specificity	97.4% (86.5-99.9%)	97.4% (86.5-99.9%)
**CA125**		
Sensitivity	75.0% (53.3-90.2%)	83.3% (58.6-96.4%)
Specificity	87.2% (72.6-95.7%)	87.2% (72.6-95.7%)
**Combined**		
Sensitivity	83.3% (62.6-95.3%)	94.4% (72.7-99.9%)
Specificity	87.2% (72.6-%)	87.2% (72.6-95.7%)
Difference sensitivity to CA125 alone	8.3% (-16.3-33.0%)	11.1% (-12.2-34.4%)
Difference specificity to CA125 alone	0.0% (-16.4-16.4%)	0.0% (-16.4-16.4%)

Supplementary Table 6. Sensitivity and specificity of the WID™-cfOC score, CA125, or combination of both in samples in the early detection set samples with lower than median gDNA contamination for which CA125 were available.

	**All cancers (95% CI)**<br>10 cancers<br>16 controls	**High-risk cancers (95% CI)**<br>9 cancers<br>16 controls
**WID™-cfOC**		
Sensitivity	20.0% (2.5-55.6%)	22.2% (2.8-60.0%)
Specificity	100.0% (79.4-100.0%)	100.0% (79.4-100.0%)
**CA125**		
Sensitivity	40.0% (12.2-73.8%)	44.4% (13.7-78.8%)
Specificity	100.0% (79.4-100.0%)	100.0% (79.4-100.0%)
**Combined**		
Sensitivity	40.0% (12.2-73.8%)	44.4% (13.7-78.8%)
Specificity	100.0% (79.4-%)	100.0% (79.4-%)
Difference sensitivity to CA125 alone	0.0% (-43.6-43.6%)	0.0% (-46.0-46.0%)
Difference specificity to CA125 alone	0.0% (-14.6-14.6%)	0.0% (-14.6-14.6%)

Supplementary Table 7. Evaluation of combined WID™-cfOC and CA125 scoring by stage. Samples positive for either WID™-cfOC or CA125 (or both), were considered positive.

	**Diagnostic set**			**Early detection set***		
WID™-cfOC or CA125	**Total**	negative	positive	**Total**	negative	positive
**Stage, n**						
I	2	2	0	1	1	0
II	1	0	1	2	1	1
III	9	1	8	5	2	3
IV	6	0	6	0	0	0
Unknown	6	1	5	1	1	0
**Total, n**	24	4	20	9	5	4

* including only samples with lower than median gDNA contamination.

Supplementary Figures

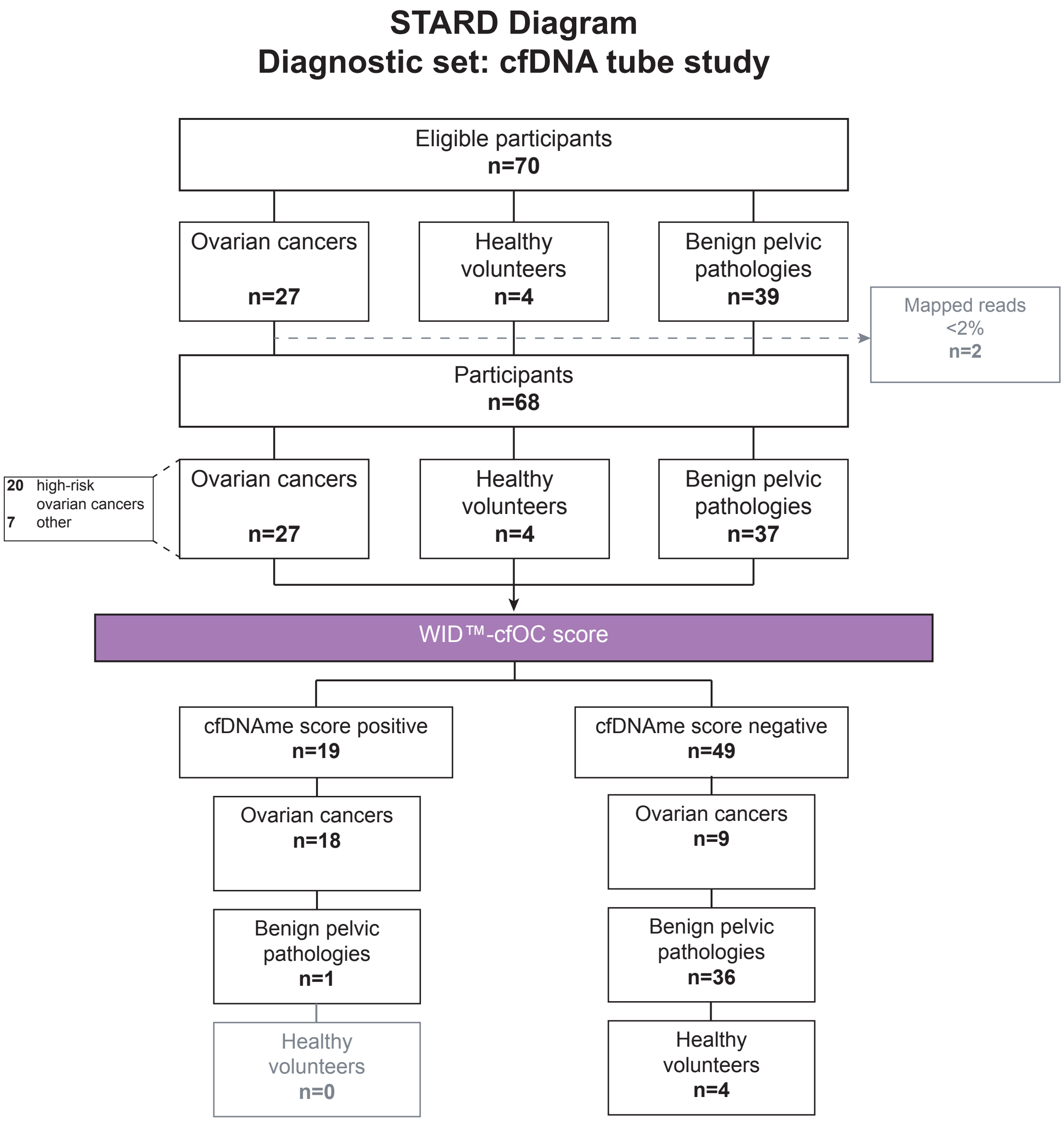

Supplementary Figure 1. STARD Diagram of the diagnostic set (cfDNA tube study).

STARD Diagram
Early detection set: UKFOCSS

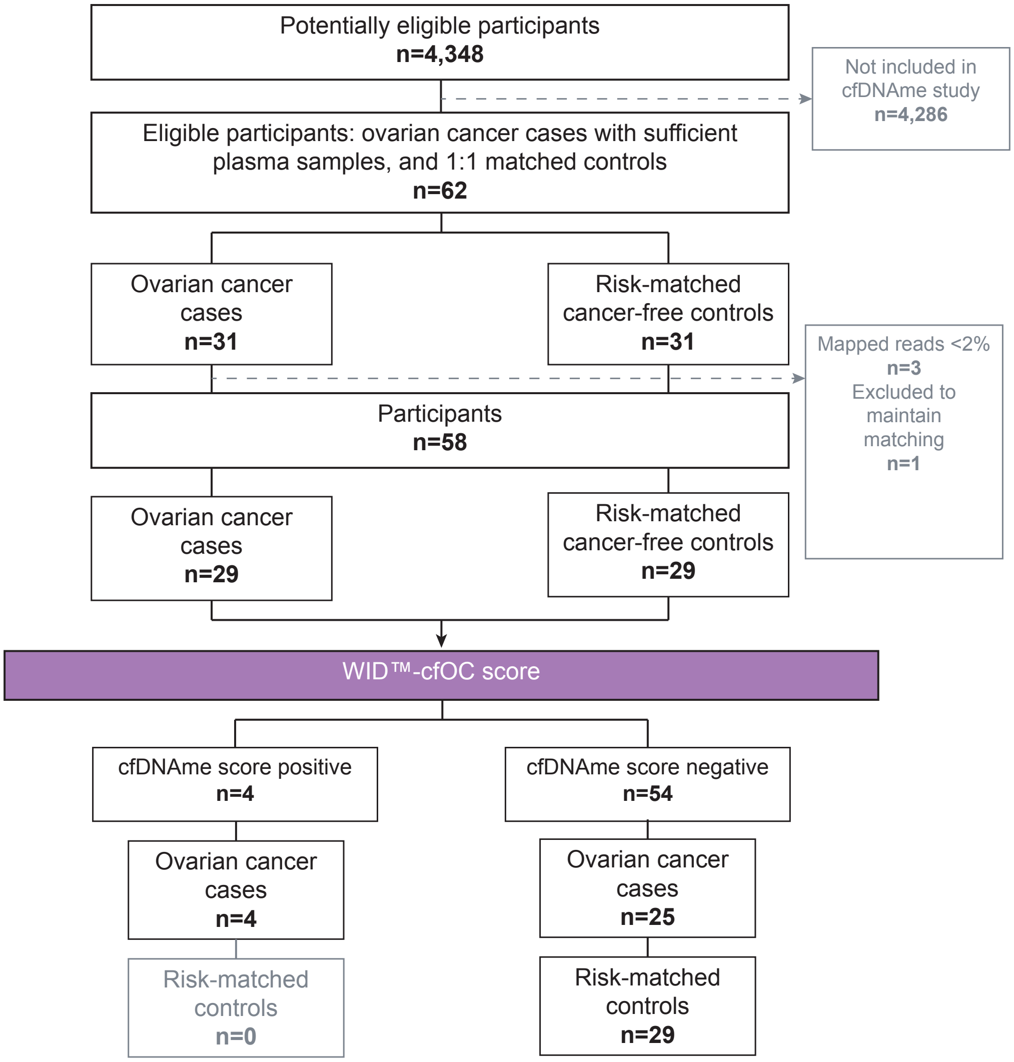

Supplementary Figure 2. STARD Diagram of the early detection set (UKFOCSS).

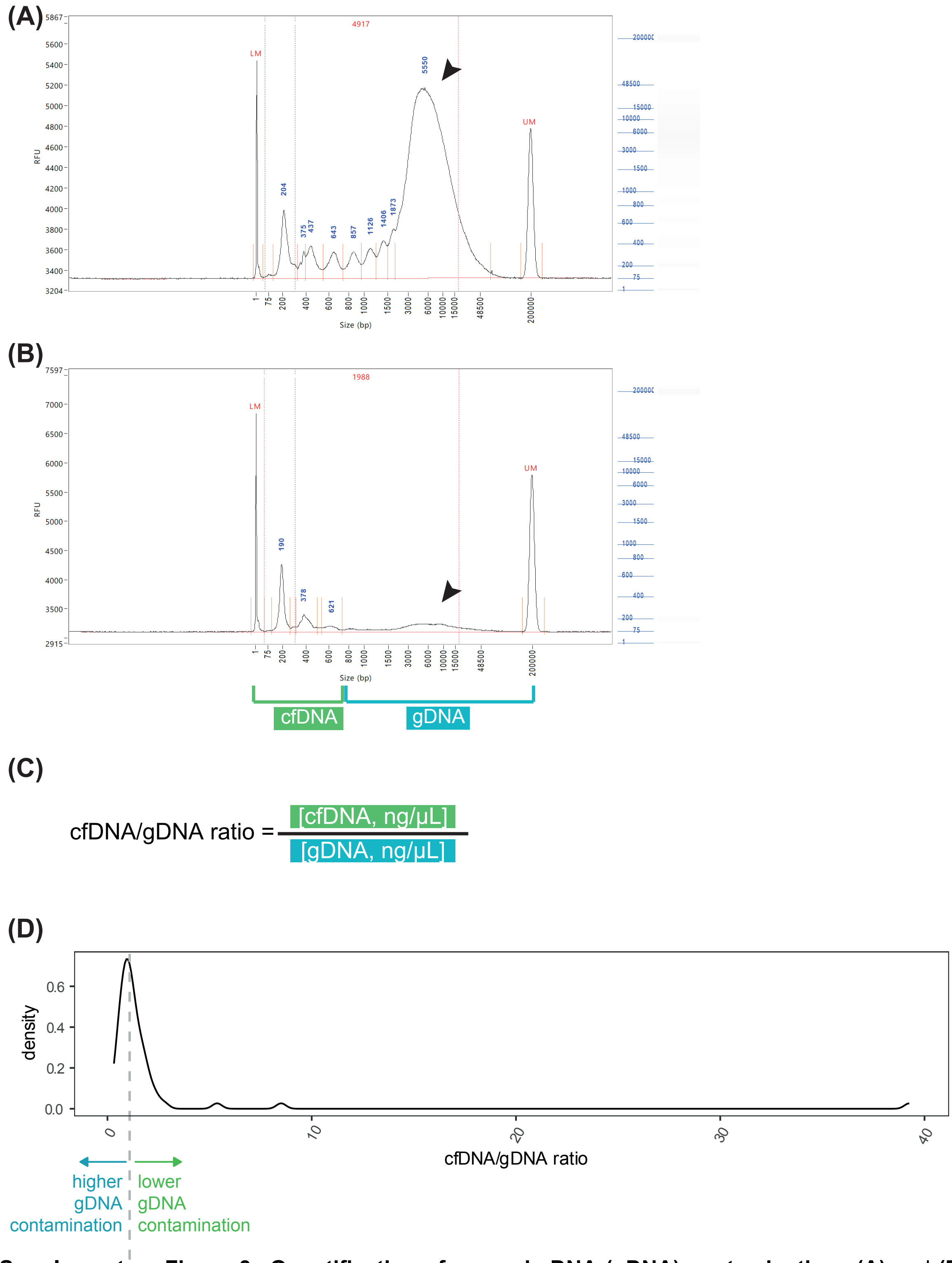

Supplementary Figure 3. Quantification of genomic DNA (gDNA) contamination. (A) and **(B)** show DNA size distributions of two exemplary UKFOCSS samples with high and lower gDNA contamination, respectively. Black arrow shows gDNA contamination peak. **(C)** For quantification of gDNA contamination, the ratio of cfDNA concentration (ng/uL) to gDNA (ng/uL) was computed. **(D)** The median cfDNA/gDNA ratio (dashed grey line) was defined as the cutoff for stratification, with a cfDNA/gDNA ratio higher than the median indicating a lower relative gDNA contamination and vice versa.

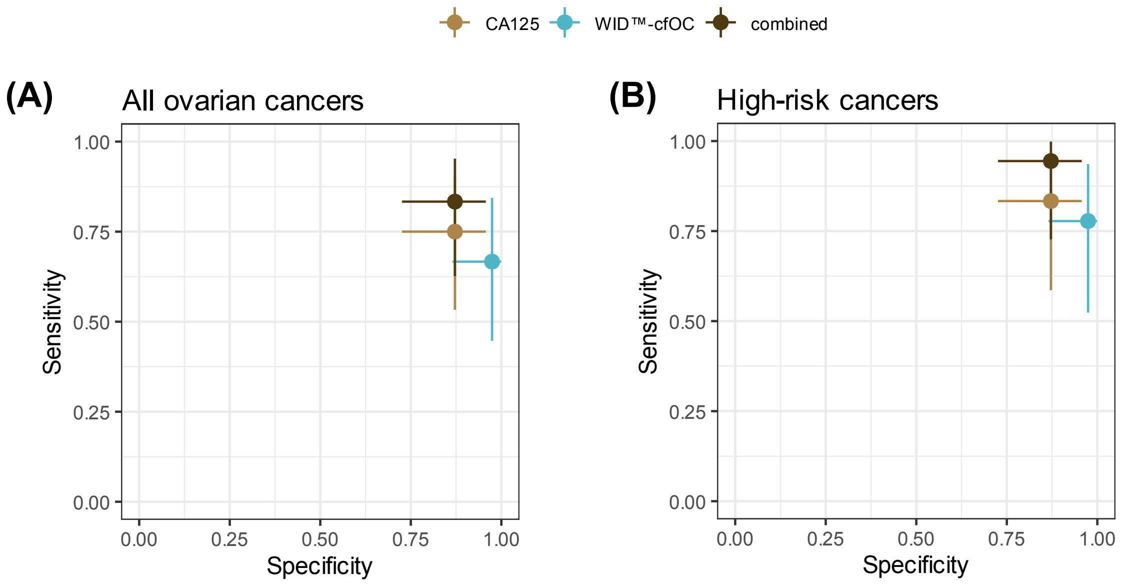

(C) WID™-cfOC score in CA125 negative samples

	OC	Control	All
**WID™-cfOC positive**	2	0	2
**WID™-cfOC negative**	4	34	38
**Total**	6	34	40
**Sensitivity | Specificity**	33.3%	100%	
**95% CI**	4.3-77.7%	89.7-100%	

(D) WID™-cfOC score in CA125 negative samples (cases: high-risk cancers only)

	OC	Control	All
**WID™-cfOC positive**	2	0	2
**WID™-cfOC negative**	1	34	35
**Total**	3	34	37
**Sensitivity | Specificity**	66.7%	100%	
**95% CI**	9.4-99.2%	89.7-100%	

(E) WID™-cfOC score in CA125 positive samples

	OC	Control	All
**WID™-cfOC positive**	14	1	15
**WID™-cfOC negative**	4	4	8
**Total**	18	5	23
**Sensitivity | Specificity**	77.8%	80%	
**95% CI**	52.4-93.6%	28.4-99.5%	

(F) WID™-cfOC score in CA125 positive samples (cases: high-risk cancers only)

	OC	Control	All
**WID™-cfOC positive**	12	1	13
**WID™-cfOC negative**	3	4	7
**Total**	15	5	20
**Sensitivity | Specificity**	80%	80%	
**95% CI**	51.9-95.7%	28.4-99.5%	

Figure 4. Comparison of CA125 and WID™-cfOC in the Diagnostic set. Sensitivity and specificity of two scores separately, or a combined score that was positive if either one of the two tests was positive, are shown for **(A)** all cancers (n=24), or **(B)** high-risk cancers (n=18) versus controls (healthy controls, n=4, benign controls, n=35). Error bars indicate 95% confidence intervals. **(C), (D)** indicate how the sensitivity and specificity of the WID™-cfOC in CA125-negative samples (including all or only high-risk cancers as cases). CA125 levels $\geq$ 35 units/mL were deemed positive. **(E), (F)** indicate the sensitivity and specificity of the WID™-cfOC in CA125-positive samples (including all or only high-risk cancers as cases).

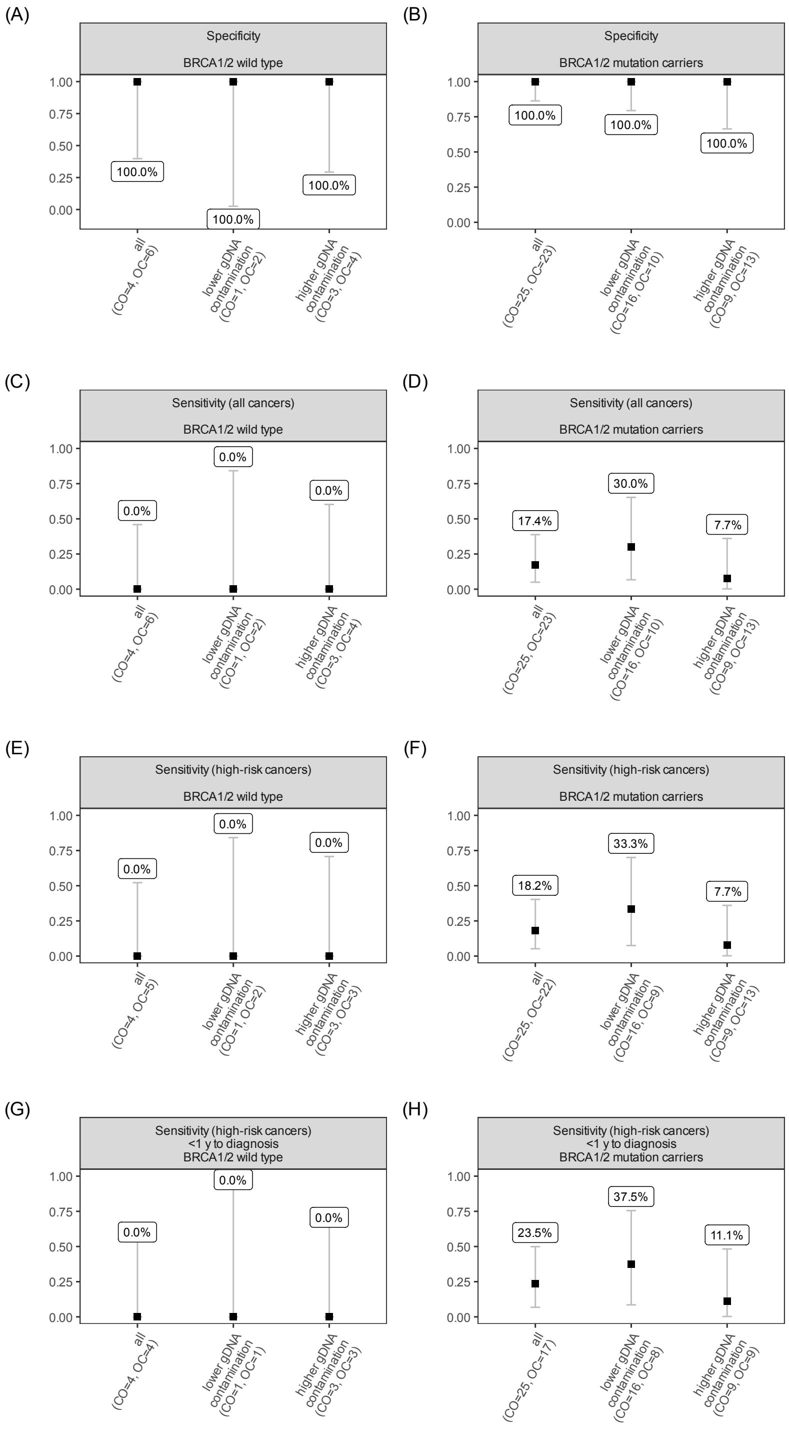

Supplementary Figure 5. Sensitivity and specificity by *BRCA1/2* mutation status in the early detection set. (A) Specificity of the WID™-cfOC in all samples, samples with lower than median gDNA contamination, or higher than median gDNA contamination, from women without *BRCA1/2* mutation or women **(B)** with *BRCA1/2* mutation. **(C)** Sensitivity of the WID™-cfOC to detect any cancer in all relevant samples, samples with lower than median gDNA contamination, or higher than median gDNA contamination, from women without *BRCA1/2* mutation or women **(D)** with *BRCA1/2* mutation. **(E)** Sensitivity of the WID™-cfOC to detect high-risk cancers in all relevant samples, samples with lower than median gDNA contamination,

or higher than median gDNA contamination, from women without *BRCA1/2* mutation or women **(F)** with *BRCA1/2* mutation. **(G)** Sensitivity of the WID™-cfOC to detect high-risk cancers within 1 year of diagnosis in all relevant samples, samples with lower than median gDNA contamination, or higher than median gDNA contamination, from women without *BRCA1/2* mutation or women **(H)** with *BRCA1/2* mutation.

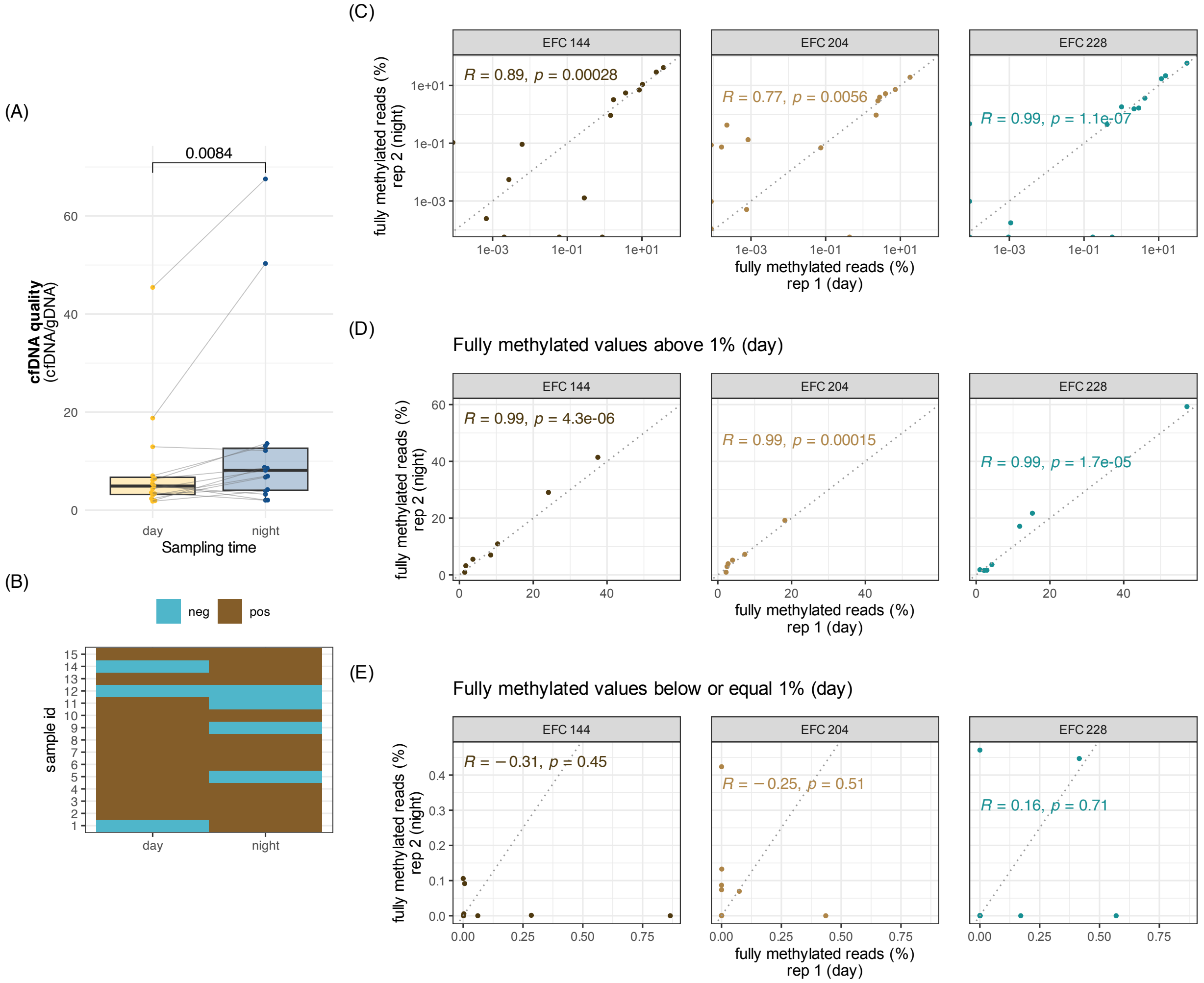

Supplementary Figure 6. Precision of the assay as evaluated in a circadian analytical assessment set. (A) Paired values of cfDNA quality (cfDNA/gDNA ratio) for samples from day and night. **(B)** Score calls for the WID™-cfOC in matched samples. **(C)** Correlation of values across day and night (logarithmic scale to account for low and high values). **(D)** Correlation of values across day and night for those samples where the daytime value yielded a fully methylated percentage higher than 1%. **(E)** Correlation of values across day and night for those samples where the daytime value exhibited a fully methylated percentage below or equal to 1%.
